# Assessment of non-invasive techniques and herbal-based products on dermatological physiology and intercellular lipid properties

**DOI:** 10.1016/j.heliyon.2020.e03955

**Published:** 2020-05-25

**Authors:** Nor Hazwani Mohd Ariffin, Rosnani Hasham

**Affiliations:** a Institute of Bioproduct Development, Universiti Teknologi Malaysia, 81310 Johor Bahru, Johor, Malaysia; b Department of Bioprocess and Polymer Engineering, School of Chemical and Energy Engineering, Faculty of Engineering, Universiti Teknologi Malaysia, 81310 Johor Bahru, Johor, Malaysia

**Keywords:** Biochemistry, Bioengineering, Biological sciences, Biotechnology, Plant biology, Non-invasive, Skin biophysical, Epidermal lipid, Skin barrier, Herbal plants

## Abstract

Skin is the largest external organ of the human body. It acts as a barrier to protect the human body from environmental pollution, mechanical stress, and excessive water loss. The defensive function resides primarily on top of the epidermis layer commonly known as stratum corneum (SC). Human SC consists of three major lipids, namely ceramide, free fatty acid, and cholesterol that comprise approximately 50%, 25%, and 25% of the total lipid mass, respectively. The optimal composition of SC lipids is the vital epidermal barrier function of the skin. On the other hand, skin barrier serves to limit passive water loss from the body, reduces chemical absorption from the environment, and prevents microbial infection. In contrast, epidermal lipids are important to maintain the cell structure, growth and differentiation, cohesion and desquamation as well as formation of a permeability barrier. Multiple non-invasive *in vivo* approaches were implemented on a regular basis to monitor skin physiological and intercellular lipid properties. The measurement of different parameters such as transepidermal water loss (TEWL), hydration level, skin elasticity, collagen intensity, melanin content, sebum, pH, and tape stripping is essential to evaluate the epidermal barrier function. Novel non-invasive techniques such as tape stripping, ultrasound imaging, and laser confocal microscopy offer higher possibility of accurate and detailed characterisation of skin barrier. To date, these techniques have also been widely used to determine the effects of herbal plants in dermatology. Herbal plants have been traditionally used for ages to treat a variety of skin diseases, as reported by the World Health Organisation (WHO). Their availability, lower cost, and minimal or no side effects have created awareness among society, thus increase the demand for natural sources as the remedy to treat various skin diseases. This paper reviews several non-invasive techniques and evaluations of herbal-based product in dermatology.

## 1 Introduction

Non-invasive procedures can be defined as treatment without incision into the skin and contact with a mucous membrane or internal body cavity other than through a natural or artificial body orifice. The procedures and instruments are classified as safe and simple. The term “non-invasive” can be translated as “no harm”, “no contact”, “no alteration of structure or function”, and “maintaining integrity of organism” [1]. Therefore, non-invasive can be literally interpreted as “a procedure or instrument that causes minimal and temporary changes to structure or function, such as painless, without incision or blood loss” [1]. Nowadays, the non-invasive techniques have great benefits and huge capabilities in determining the skin's physiological and intercellular properties especially for characterization of skin barrier.

Skin intercellular lipid characterises and determines the profiling of lipid species in the biological system which is highly associated with skin type. Skin barrier mainly comprises of corneocytes and a lipid-enriched intercellular matrix. Ceramide is the major lipid found in SC, with 50% abundancy, followed by free fatty acid (25%) and cholesterol (25%) [2]. These extracellular lipids are secreted from lamellar bodies (LB) into the intercellular space of SC. LB contain phospholipids, spingomyelin, glucosylceramide, and cholesterol, which are metabolised by enzymes and secreted into intercellular lipids [3].

The epidermal barrier plays an important role in the development of atopic eczema (AE), while skin lipids contribute to barrier integrity. It limits passive water loss, reduces chemical absorption from the environment, and prevents microbial infection. The defensive function resides primarily in the upper part of the epidermis, whereby the skin barrier is integrated with the SC formation and homeostasis. For example, the decrease of SC hydration and permeability alteration of barrier functions could lead to various skin disorders, such as atopic dermatitis, psoariasis, and ichthyosis [4, 5, 6]. That being said, a proper development and maintenance of SC is the key to its remarkable ability to defend the body against both chemical and microbial attacks as well as dehydration [7].

Non-invasive techniques have been established for centuries to determine the herbal plant efficacy in treating skin diseases. Nowadays, the application of herbal products has become a natural approach to a healthy lifestyle [8]. The fact that natural remedies are more reliable and efficient to treat skin diseases with minimal or no side effects as compared to other conventional drugs draw massive attention in dermatology study [9, 10, 11]. Owing to the increasing cost of maintaining personal health, natural remedies have become more common to treat minor ailments [12], thus becomes the major reason for the increasing demands for natural-based remedy in Asia, especially China (Wu-Hsing), India (Aryuvedic, Unani, Siddha), and Japan (Kampo) [13, 14, 15].

Herbs have been classified as potential agricultural commodities under the National Key Economic Area (NKEA) and are expected to contribute to the country's income and create employment opportunities. For example, Mas Cotek (*Ficus deltoidea*), Misai Kucing (*Ortho siphon aristatus/stamineus benth*), Lidah Buaya (*Aloe Vera Inn*), and Tongkat Ali (*Eurycoma longifolia*) are potential herbal crops for medicinal use. In Malaysia, the herbal industry has a great potential to encourage the national tourism and business development, especially in pharmaceutical and cosmeceutical industries [8].

However, herbal medicinal must fulfil the technical safety and application standards (norms) required by society. Both local and international products are regulated under Sale of Drugs Act 1952 (Revised 1989) and Control of Drugs and Cosmetics Regulations 1984 (amended 2009). This paper focused on non-invasive techniques to assess skin physiology and intercellular lipid properties which is correlated to skin physiological conditions and epidermal lipid profiles. The efficacy of herbal plants using the non-invasive techniques to treat skin diseases was briefly discussed. Literature was obtained from the following database: Science Direct, PubMed, Google Scholar, and Springer Link for scientific publications.

## 2 Epidermal skin structure

The epidermis is the outermost layer of the skin. In general, epidermis consists of basal layer (source of replacement cell), spinous layer (centre of the epidermis where keratinocytes make keratin), granular cell layer (site of water barrier), and stratum corneum (thick keratinised outer layer which prevents water loss and provides anti-trauma and anti-infectious barrier). It is made up of 95% keratinocytes, Langerhans cells, Merkel cells, inflammatory cells as well as melanocytes. Melanocytes (found in the basal layer) are specialised neural crest cells which produce melanin, a protective pigment that absorbs harmful UV radiations and produces energy as harmless heat through a route referred to as ‘ultrafast internal’. Langerhans cells are the immune cells responsible in antigen presentation, which literally assist the skin's immune system. Merkel cells found in the basal layer are associated with sensory nerve endings [16].

The skin is responsible to guard underlying muscles, bones, ligaments, and internal organs. There are two general types of skin, namely hairy and glabrous skin [17]. However, the skin can be dry, sensitive, pale, sagging, or tired. Individuals who suffer from beta-carotene, B complex, vitamins C and E deficiency often encounter dry skin problems. Considering the fact that skin interfaces with the environment, skin plays a key role in protecting the body against pathogens [18, 19] and excessive water loss [19]. Besides, skin also plays an important role in insulation, temperature regulation, sensation, storage, the synthesis of vitamin D by the action of UV, the protection of vitamin B folates, the absorption of oxygen and drugs [20], and water resistance.

## 3 The non-invasive assessment of skin physiological conditions

The Multi Probe Adapter (MPA) system is used to measure skin biophysical properties. The modular system is a basic device equipped with specific digital probes that can be adjusted according to the user preferences. Calibration data are stored inside the system itself. The advantage of using this method is that the probes can be connected to any independent devices and simultaneously transmit data to related software. The probes can usually measure transepidermal water loss, hydration, melanin, erythema, elasticity, collagen, sebum, and pH of the skin.

### 3.1 Skin transepidermal water loss (TEWL)

Transepidermal water loss (TEWL) was measured regularly in order to provide further information on the epidermal permeability barrier—either normal, experimentally perturbed, or in diseased conditions [21]. Low TEWL values are a basic feature of in vivo intact skin function [21, 22]. Elevated TEWL values indicate the skin barrier abnormalities, which are the major reason of several diseases, such as atopic dermatitis and ichthyosis vulgaris [23, 24, 25, 26, 27, 28].

**Figure 1:**
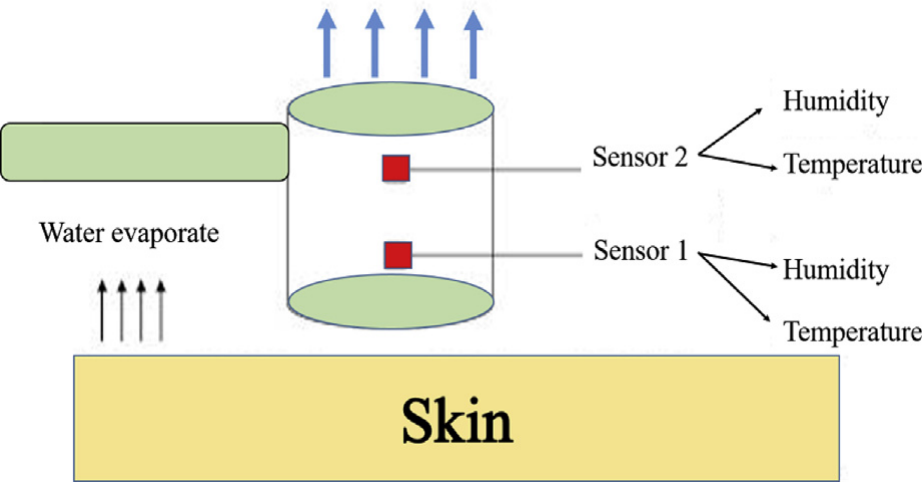
Illustration of the measurement principle, Tewameter TM 300 (Modified figure from) [30]).

TEWL can be measured by evaporimeter (Tewameter® TM 300; Courage & Khazaka). Figure 1 illustrates the measurement principle, Tewameter® TM 300. Tewameter was particularly designed according to Nilsson's Vapour Pressure Gradient theory, with an open chamber method that provides minimal impact on the skin being examined with low statistical bias. The system consists of a hollow cylinder with two hygroscopic and temperature sensors to measure the density gradient of water evaporation pressure at different areas on the skin surface. The differences between the two measurements points are calculated by Fick's laws of diffusion in grams per hour per square meter (g/h/m^2^), as stated in Eq. (1) [29]. However, the horny layer is not an inert membrane, but shows some affinity to water. Therefore, Fick's Law can be modified by the introduction of a partition coefficient Km [1]. Fick's law concept is a diffusive mass that will move from a region of high concentration to a region of low concentration across a concentration gradient.

(1) $$\text{Km}=\frac{\left(\text{Water}\;\text{concentration}\;\text{in}\;\text{the}\;\text{lower}\;\text{horny}\;\text{layer}\right)}{\left(\text{Water}\;\text{concentration}\;\text{in}\;\text{the}\;\text{intercellular}\;\text{space}\;\text{of}\;\text{living}\;\text{epidermis}\right)}$$

Fickʼs laws of diffusion suggest that the diffusion rate of gas across a permeable membrane can be evaluated by several factors, such as the chemical nature of the membrane, surface area, thickness, and partial pressure gradient of the gas (Table 1).

**Table 1:** Interpretation of TEWL results [23].

TEWL – values g/h/m^2^	Interpretation
0–10	Very healthy condition
10–15	Healthy condition
15–25	Normal condition
25–30	Strained skin
Above 30	Critical condition

### 3.2 Skin hydration

SC hydration is another important parameter that can be linked to assess epidermal functions. A variety of instrument- and environment-related variables, such as ambient air temperature, relative air humidity, and direct air flow may affect the hydration measurement. The factors originating from an individual include the age, sex, anatomic site, sweat, and skin surface temperature, which may influence the barrier-related parameters [29, 32].

**Figure 2:**
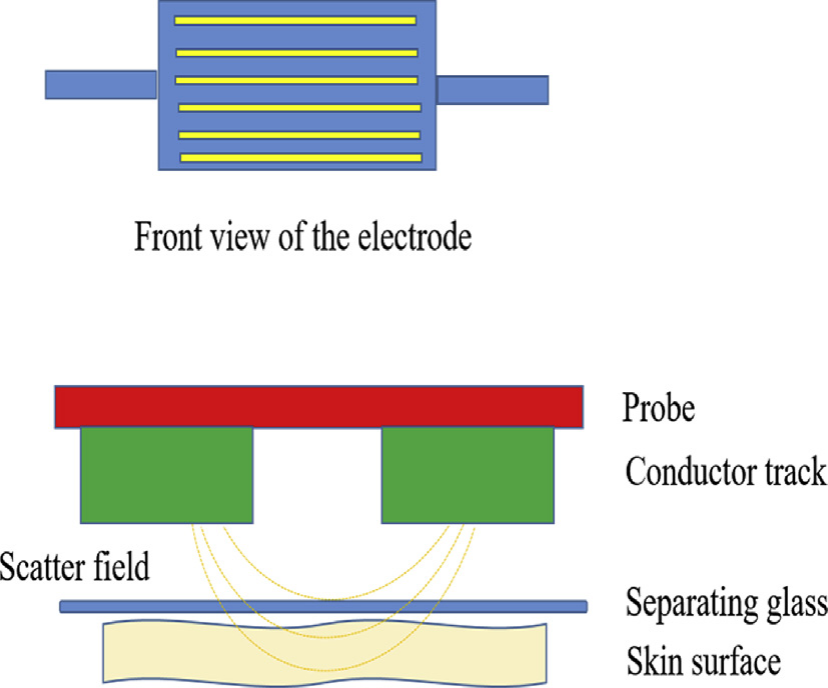
Illustration of the measurement principle, Corneometer® CM 825 (Modified figure from [34]).

The hydration level of skin surface can be accurately determined using Corneometer® CM 825 (Courage & Khazaka) by measuring electrical capacity as the alternating voltage of SC. Figure 2 shows the measurement principle of Corneometer® CM 825. The higher the water content in epidermis, the higher is its electrical capacity [33], resulting in higher value of SC. Adequate skin hydration is vital to maintain a healthy skin, which makes moisturiser an important component in basic skin care. Table 2 below represents the interpretation of skin hydration.

**Table 2:** Interpretation of skin hydration results [23].

Moisture value	Body Parts	
	Forehead, cheek, chin	Hand, arms
Very dry	<30	<5
Dry	30–60	5–25
Sufficiently moisturized	>60	>25

### 3.3 Skin melanin and erythema

**Figure 3:**
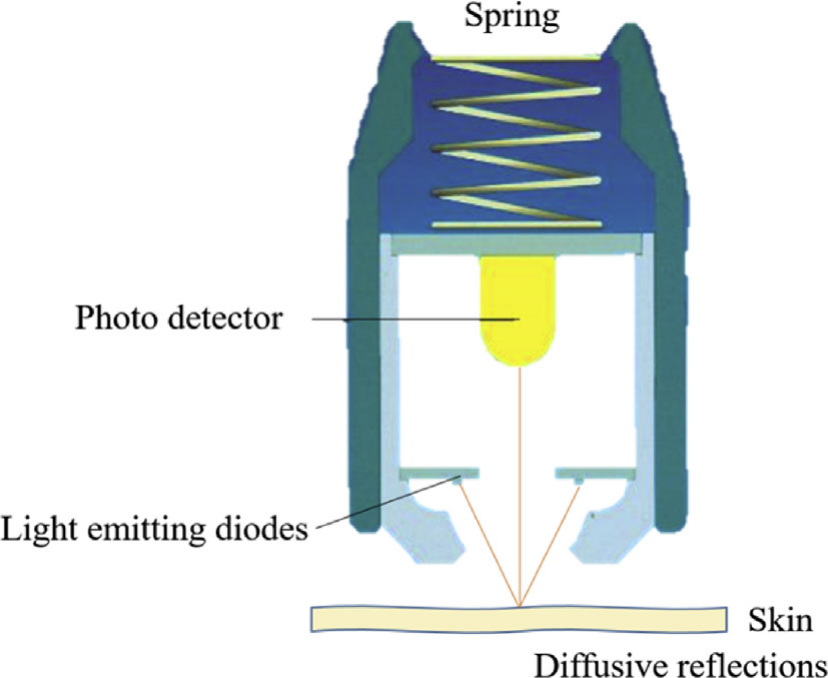
Illustration of the measurement principle, Mexameter® MX18 (Modified figure from [36]).

Skin colour is predominantly determined by pigments such as hemoglobin, bilirubin carotene and mostly, melanin. Melanin is the main characteristic to differentiate ethnic types. Skin pigmentation primarily evolves to regulate UV radiation, penetrating skin by controlling its biochemical effects and can be significantly altered by substances, such as drugs and irritants [35]. Figure 3 illustrates the measurement principle of Mexameter® MX18.

Erythema is highly associated to skin redness. It occurs along with skin injury, inflammation, or infection. In general, erythema can be caused by infection, acne medication, exercise, massage, allergies, solar radiation (sunburn), cutaneous radiation syndrome (acute radiation exposure to skin) leading the capillaries in the skin to dilate (hypereremia), resulting in skin redness [37].

Mexameter® MX18 is a device to measure the quantities of two major components responsible for skin colour, namely melanin and hemoglobin (erythema). The measurement is based on the absorption and reflection of an active colour detecting chip. Briefly, melanin is measured using two wavelengths that are chosen according to different absorption peaks of melanin pigments, while erythema measures are used to estimate the redness level (hemoglobin) in skin.

### 3.4 Skin elasticity

**Figure 4:**
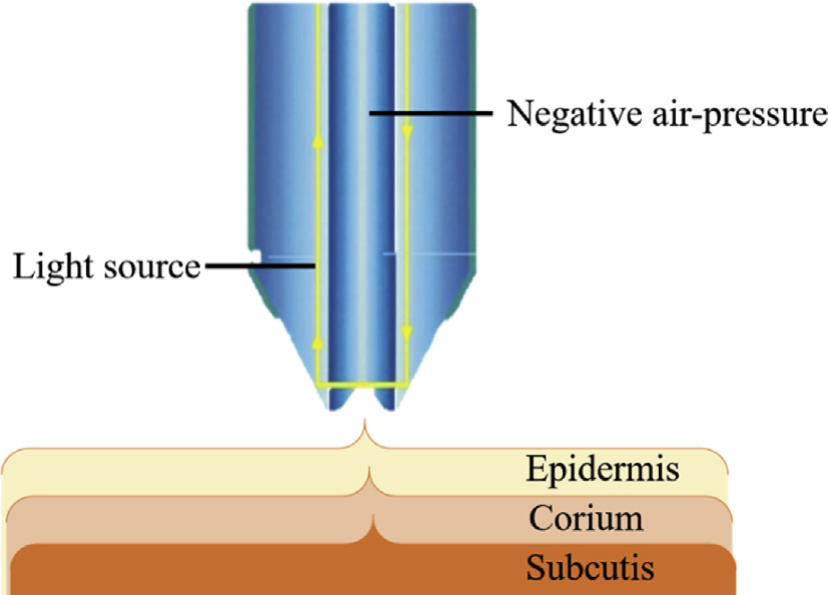
Illustration of the measurement principle, Cutometer (Modified figure from [39]).

The mechanical properties of skin can be assessed by evaluating the thickness and qualitative properties of epidermis, dermis, and subcutis. Aging causes qualitative and quantitative changes in skin, such as loss of elasticity, reduction in the epidermal thickness and collagen content, increased production of wrinkles as well as pigment lesions. However, these features may vary among individuals [38]. Figure 4 illustrates the measurement principle of Cutometer®.

**Figure 5:**
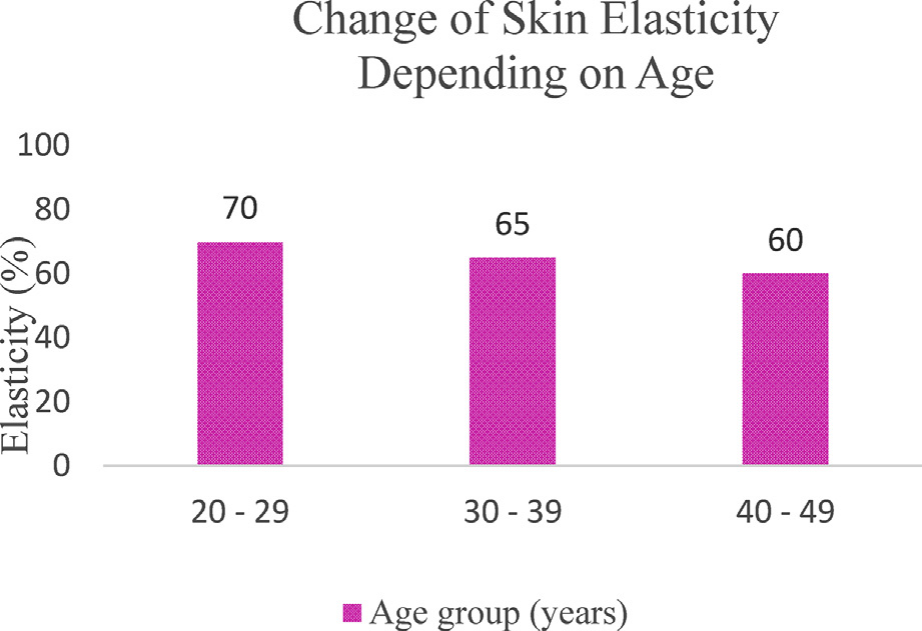
Change of skin elasticity depending on age groups [31].

Skin elasticity is measured by suction with respective probes according to Nilsson's Vapour Pressure Gradient Method with Cutometer® (Courage & Khazaka) probes. The Cutometer® is designed to measure the elasticity of the upper skin layer using negative pressure which mechanically deforms the skin. Suction is generated to produce negative pressure which consequently draws the skin into the aperture of the probe. The penetration depth inside the probe is evaluated by a non-contact optical measuring system. Figure 5 shows the skin elasticity changes which are categorised based on age.

### 3.5 Skin collagen

Collagen is a type of protein manifested in the skin, bone, tendon, cartilage, and blood vessels. They are predominantly rich in glycine, proline, and hydroxyproline [40]. Human dermis consists primarily 70% collagen to provide good support, maintain elasticity and tensile as well as to reinforce skin structure in order to appear smooth and young.

**Figure 6:**
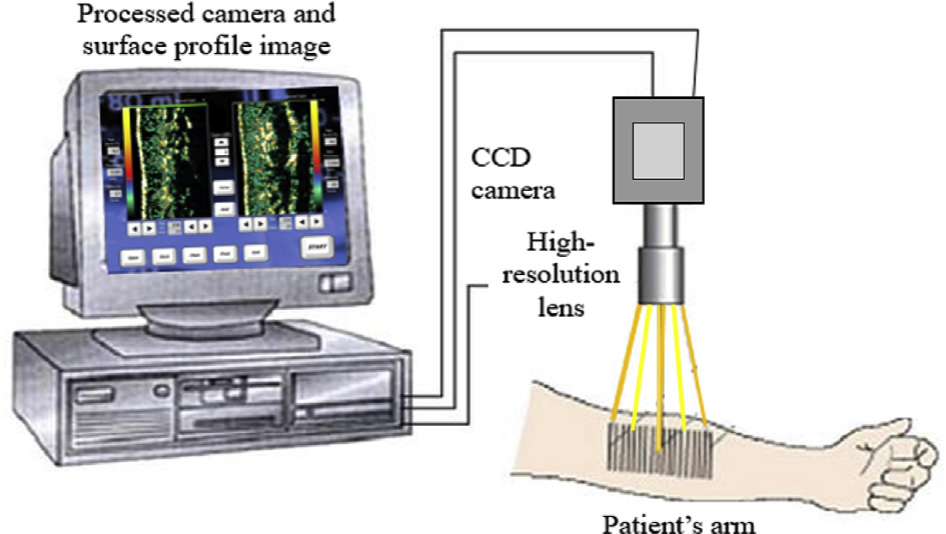
Illustration of the measurement principle using ultrasound skin imaging (Modified figure from [41]).

Skin high resolution ultrasound by DermaLab® Combo (Cortex Technology) has been widely employed for skin scanning. Ultrasonic imaging relies on the properties of reflected sound waves through the tissue. Different tissues reflect waves distinctively due to the variations in tissue structure, vascularity, and density, which are highly correlated to the differences of collagen, keratin, and water content [41]. Translating this fact to dermatology, dermis that appears echogenic (transducers of 20 MHz) with echoes originating from the fibre network can be considered to comprise elastic fibres, collagen, and tissue atrophy. Figure 6 shows the illustration of the measurement principle using ultrasound skin imaging.

### 3.6 Skin sebum

**Figure 7:**
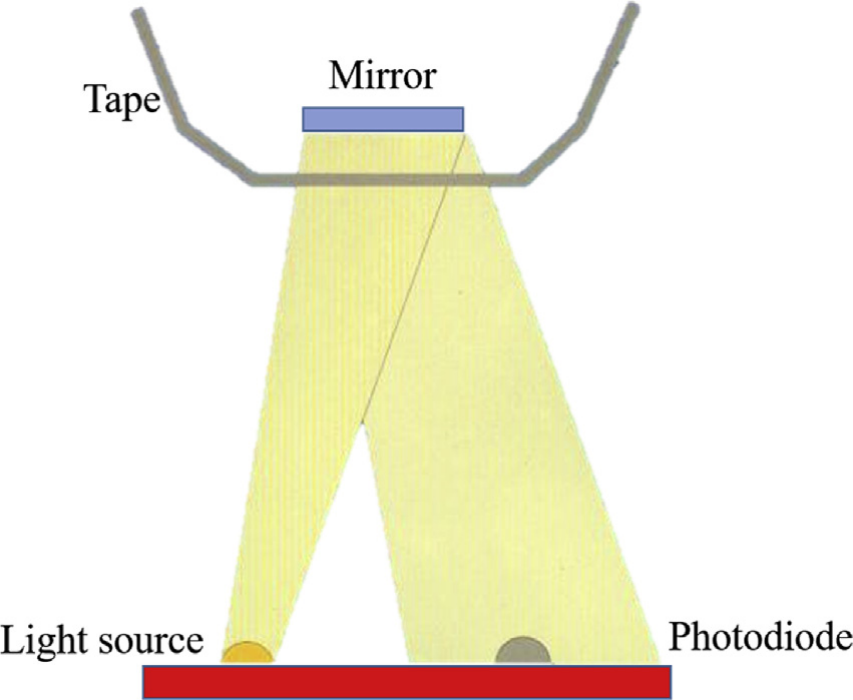
Illustration of the measurement principle, Sebumeter® SM 815 (Modified figure from [43]).

Sebum lipids on the skin surface have major impact on the protective and mechanical properties of the epidermal barrier. Sebumetry (Sebumeter® SM 815; Courage & Khazaka) is generally used to quantify the sebum production on the skin surface. In brief, a special tape will become transparent after physical contact with the sebum on the skin surface. The translucency of the tape will be measured using a photometry system. The light permeability of the tape changes after 30 s of skin contact, depending on the sebum content on the skin surface [42]. Figure 7 depicts the measurement principle of Sebumeter® SM 815. Table 3 represents the result of skin sebum interpretation.

**Table 3:** Interpretation of skin sebum results [23].

Moisture value	Body Parts	
	Forehead, cheek, chin	Hand, arms
Very dry	<30	<5
Dry	30–60	5–25
Sufficiently moisturized	>60	>25

### 3.7 Skin pH

**Figure 8:**
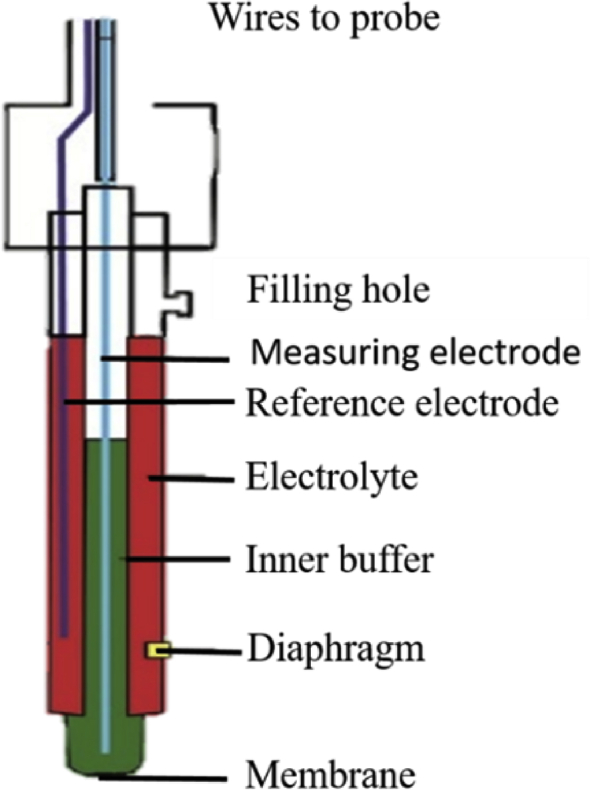
Illustration of the measurement principle, Skin pH Meter® PH 905 (Modified figure from [46]).

The pH value of skin is associated with the quality of hydrolipid film. Basically, the pH of stratum corneum regulates three epidermal functions, which are antimicrobial barrier, permeability barrier homeostasis, and barrier integrity/cohesion. The pH alterations on stratum corneum could lead to abnormal epidermal barrier function [44]. Skin pH Meter® PH 905 (Courage & Khazaka) is a probe consisting of a flat-topped glass electrode (to enhance skin contact) connected to a voltmeter which is specifically designed to determine the pH values of skin. In general, the activity of hydrogen cations is measured based on its adjacency to the thin layer of hydrated gel which is located on top of the probe [45]. Figure 8 shows the measurement principle of Skin pH Meter® PH 905. Table 4 represents the interpretation of skin pH results.

**Table 4:** Interpretation of skin pH results [23].

pH	<3.5	3.8	4.0	4.3	4.5	5.0	5.3	5.5	5.7	5.9	6.2	6.5	>6.5
Women	+ Acidic range -				Normal				- Alkaline range +				
Men	+ Acidic range -				Normal				- Alkaline range +				

## 4 The non-invasive assessment of skin intercellular lipid profiling

### 4.1 Tape stripping

Tape stripping that involves the subsequent removal of SC using adhesive tapes has emerged as a useful technique to study the physiology of SC in recent years [47, 48]. The stripping tape is placed onto the subject's skin and slowly removed. Flakes that are stuck on the tape surface can be observed under optical microscope. This method is simple, non-invasive, requires no chemical consumption, and allows the collection of different layers of corneocytes separately (via sequential application of the tape to the same area of the skin) [49].

Tape stripping is a universal method and can be applied in *in vivo* [50, 51, 52] and *in vitro* studies [53, 54, 55]. The amount of SC removed by a single adhesive tape strip depends on several intrinsic factors, such as the number of cell layers [56] and corneocytes [57], the thickness of SC [58] as well as the composition and amount of lipids [59]. The amount of SC removed varies on the anatomical site. Tape stripping method is a relatively fast and simple technique, which is suitable for large-scale studies in humans [60].

Previous studies conducted on the different layers of SC demonstrate a significant increase of phospholipids and marked decrease in total ceramides as compared with more superficial ones [61]. In contrast, Weerheim and Ponec [62] found no ceramide gradient using the same method in healthy volunteers. However, it can be concluded that the ratio of cholesterol/ceramide in the inner and outer SC has no significant difference [63]. Denda *et al.* studied the influence of tape stripping on the quantitative amount of lipids in SC reported an increase in ceramides 1 and 2 and a decrease in other types of ceramides, which clarify the way in which these substances affect scaly skin [64].

**Figure 9:**
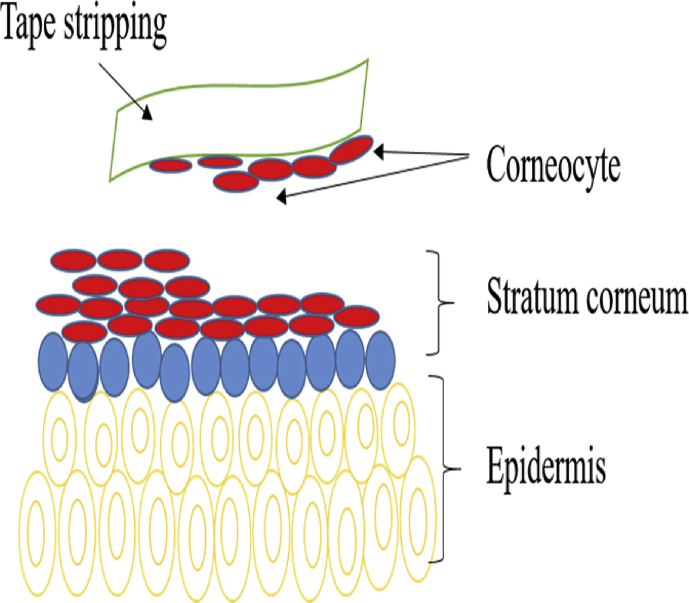
Schematic of the tape stripping test (Modified figure from [70]).

The amount of protein is directly correlated to the amount of SC removed using tape stripping [65, 66]. The amount of SC removed by each strip literally decreased as the SC is progressively stripped [67, 68, 69]. The adhesion properties of the adhesive and the cohesiveness of the corneocytes strips will determine the removed amount of SC mass. Approximately, one-third of strips amount is needed to remove 45–50% of SC [60]. Figure 9 shows the schematic of the tape-stripping test.

### 4.2 Ultrasound

In recent years, the applications of ultrasound in dermatology have attracted a lot of attention due to the development of machines that work with high- and multiple-frequency of probes to allow the optimal definition of superficial structures [71]. Ultrasound is one of the non-invasive methods in skin imaging technologies which can be applied to objectify the shape and size of any structure. Ultrasound is a simple and reproducible technique to measure the skin thickness [72], inflammatory conditions, tissue edema, and the extent of dermal and subcutaneous fibrosis as well as to monitor the course of wound healing [73, 74, 75]. Only ultrasound with 50MHz high resolution transducer can determine the image of the epidermis [76]. The ideal morphology of the skin should require the absence of contact between the device and the skin, the images recorded at video rate, a depth of the skin thickness, spatial resolution, and a volume vision [77].

Moreover, high variable-frequency ultrasound is an advanced technique that produces quantitative and qualitative information on the skin lesions and surrounding tissues. It is capable to define deeper structures of the skin layers and perfusion patterns in real time. A previous study by Wortsman and Wortsman reported that skin ultrasound is a highly effective adjuvant to diagnose skin lesions by clearly separating the lesional from the extralesional areas, exogenous from the endogenous components, and dermatologic from the nondermatologic conditions. Therefore, it is safe to say that non-invasive ultrasound imaging provides highly relevant clinical information that can be a fundamental technique to study human skin [78].

**Figure 10:**
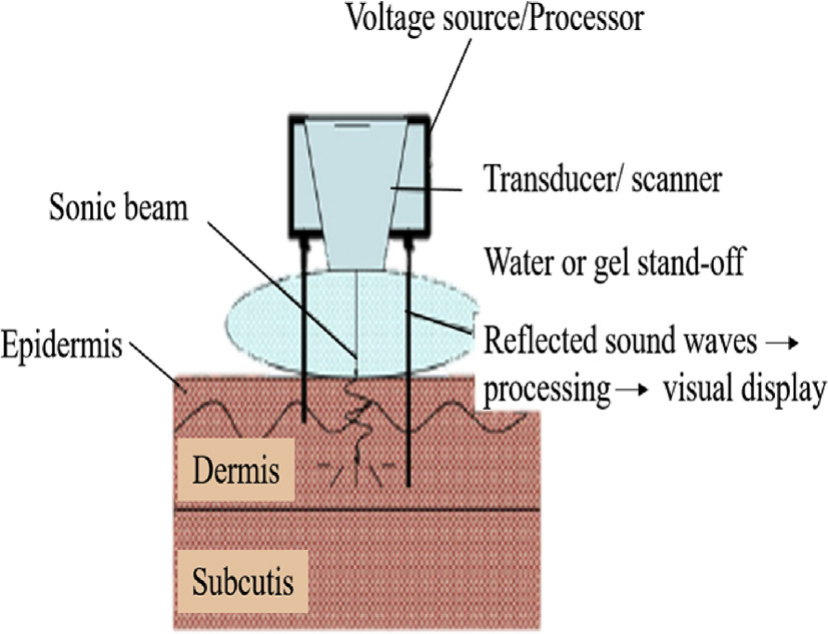
Depiction of ultrasound device, including voltage source, transducer, water or gel standoff, and sonic beam projected into skin (Modified figure from [81]).

Ultrasound was first applied in dermatology as a fixed-frequency equipment (20–100 MHz) that was able to distinguish the layers of skin; hence, several studies on cutaneous pathologies have been performed using this method [79]. Ultrasound is able to provide reasonable balance between penetration and resolution, real-time capability as well as the possibility to identify and measure both texture and blood flow changes [80]. However, despite the remarkable properties of ultrasound, it can only measure 0.1 mm lesions and detect pigments such as melanin epidermal lesions [78]. Figure 10 depicts the ultrasound device, including voltage source, transducer, water or gel standoff, and sonic beam projected into the skin.

### 4.3 Laser confocal microscopy

Confocal microscopy or known as confocal laser scanning microscopy (CLSM) was inaugurated in 1991 by New *et al.* [82]. It is a high-resolution optical detection technique [83] that provides impressive confocal images of cellular organisation in human skin [84]. The device basically visualises living epidermal cells individually and primarily focuses on natural contrast, hydration state, and environment. Besides, it is also able to measure the SC thickness at micrometer level and repair cutaneous wounds.

A number of specific interactions could occur when the laser light comes into contact with the skin surface. The light source focuses on a small volume of sample, which makes it hard and unsuitable to access a large field of view. To overcome this drawback, the confocal technique removes all focused backscattered photons from the surrounding. Laser beam that is not transmitted will be absorbed by the tissue or any material and generate heat energy which can cause thermal damage to the tissue [85]. The depth of transmission into the tissue depends on the tissue type, laser wavelength, and laser fluency [86].

**Figure 11:**
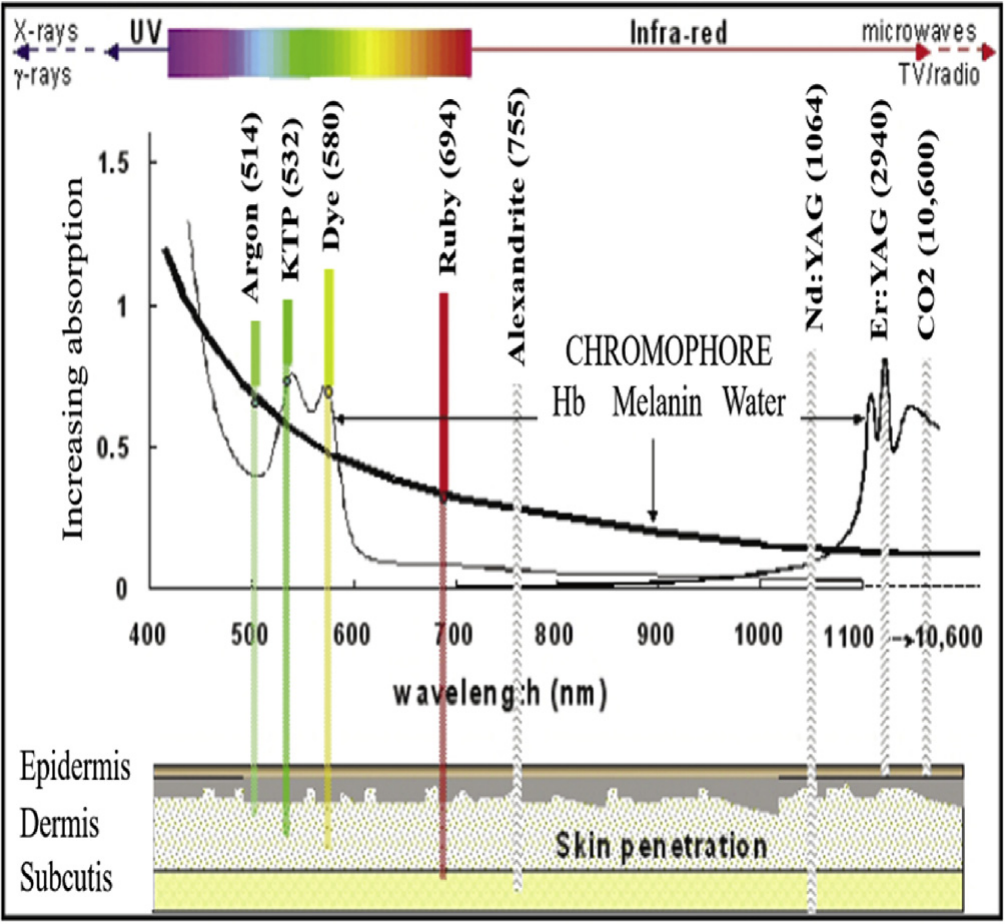
Comparative absorption spectra showing common laser wavelengths and their depth of skin penetration (Modified figure from [89]).

However, to reduce the damage from laser exposures, several preventive steps have to be considered and taken into account. For example, appropriate safety goggles must be worn to filter the specific wavelengths of laser light during an operation [87]. Cloth drapes should be wet with sterilised water or saline solution, while metal instruments are usually burnished or ebonised to decrease laser light reflection. Other than that, protective cylinders and shields should be attached to the end of the hand piece to absorb fumes, vaporised particles, and splattered blood and tissue [88]. Hazard on flammability around the treatment site is of great concern. Figure 11 shows the comparative absorption spectra along with the common laser wavelengths and their skin penetration depth.

In contrast, reflectance-mode confocal microscopy (RMCM) is a non-invasive technology with the same principle as CLSM. However, RMCM role is limited to superficial epidermal wounds as well as angiogenesis [90] and to identify skin morphology condition (normal or abnormal) [91]. To say the least, confocal microscopy potential in vivo study is very promising as compared to other modern technologies [92, 93].

## 5 The recent case study used non-invasive techniques in dermatology

The world has witnessed the application of non-invasive techniques in dermatology mainly due to their tremendous benefits and great ability to determine the skin lipid structure. However, the standardisation of non-invasive methods is a concern to get a successful output. Therefore, various factors should be taken into account to obtain reproducible and relevant results. This paper provides information that can be optimised, and thus be a great benefit to apply non-invasive techniques in dermatological studies. In contrast, to evaluate the skin functions, full range results can be embraced by employing several non-invasive techniques (Table 5). Many previous research had reported the potential mechanisms by which herbal plants can improve the skin physiological and intercellular lipid properties. The utilisation of herbal plants could be a valuable alternative approach to prevent and/or treat skin disorders. Table 5 summarised the application of non-invasive techniques in herbal plant studies.

**Table 5:** Lists of herbal based product used non-invasive techniques.

No.	Type of product	Specification herbal materials	Solvent of Extraction	Herbal Used in Product		Species/Model	Number of subjects	Mean age	References
1.	Moisturizer	Commercial products	N/A	HM1	Jojoba, vit E	Normal humans	40	40 ± 9 y/o	[111]
				HM2	Chamomilla recutita, helanthus annuus, sambucus nigra, primula veris, theobroma cacao				
				HM3	Hydolyzed elastin, talc, tocopheryl acetate				
				HM4	Aloe barbadensis				
				HM5	Elaeis guineensis, olea europaea, persa fratissima, prunus armeniaca, ribes nigrum, vitis vinifera, micro fruit oil				
				HM6	Shea butter, cocos nucifera, olea europaea fruit oil (olive), aloe barbadensis (leaf)				
				HM7	Vit E, vit A, theobroma cacao, pollen extract, triticum vulgare (wheat germ oil)				
				HM8	Cucumis sativus juice, coumarin, hexyl cinnamal, limonene				
				HM9	Aloe vera, indian madder, country mallow				
				HM10	Kapoor kachari, chandan, nimba, ghrit kumari, ushir, gulabjal, tulasi, haridra, yastimadhu, malai, grape seed oil, olive oil, badam oil, keshar, bhavpralash, tankan amla (boric acid), rastarangni				
				HM11	Santalum album (sandal wood), cuscus grass (vetiveria zizanioides), sweet basil (ocimum sanctum), aloe vera, honey				
				HM12	Behda kwath, madhu, ankurit gehum, kusumbhi tail, methi beej, vach				
				HM13	Olive oil, sesame oil, Vit E				
				HM14	Olive oil, red apple				
				HM15	Aloe vera, jojoba oil, milk cream, wheat germ				
				HM16	Vit A, D, E, Aloe vera, wheat germ oil, rose water				
				HM17	Almond, sandal wood, honey, wheat germ oil, jojoba oil, essential oil of patchouli, germanium, rose and basil				
				HM18	Grape seed, wheat germ oil, vit E, vit F				
				HM19	Cocoa butter, vit E, aloe vera extract				
				HM20	Honey, almond				
2.	Moisturizer	Different concentrations (0.135–0.9% w/w) of extracts, juice and gel	Ethanol:water	Aloe barbadensis (Leaf)		Humans with history of dry and itchy skin	20	30 ± 10 y/o	[112]
				Glycerriza glabra (Bark) Cucumis sativus (Fruit)					
				Trigonella Foenum Graecum(Seed)					
				Triticum sativum(oil)					
				Cocos Nucifera(oil)					
				Prunus Amygdalus(oil)					
				Oleum olivae(oil)					
				Azadirachta indica(Leaf)					
				Santalum Alba(oil)					
				Emblica officinale					
3.	Topical formulation	(6% w/w) Glycolic *Ginkgo biloba* extract or glycolic green tea extract	Ethanol	Ginkgo biloba, green tea		Albino hairless mice (male)	24	Not indicate	[113]
4.	Cream	3% of the concentrated extract of Basil	Ethanol	Basil		Normal humans (male)	11	48 y/o	[114]
5.	Powder (orally administered)	4.0 g Atractylodes lancea rhizome, 4.0 g Hoelen, 3.0 g Cnidium rhizome, 3.0 g Japanese Angelica root, 2.0 g Bupleurum root, 1.5 g Glycyrrhiza root, and 3.0 g Uncaria thorn.	Purified water	Yokukansan (*Atractylodes lancea* rhizome, Hoelen, *Cnidium* rhizome, Japanese Angelica root, *Bupleurum* root, *Glycyrrhiza* root, and *Uncaria* thorn)		Mice (male)	-	10 weeks	[115]
6.	Cream	5% concentrated extract of *T. chebula*	N/A	Terminalia chebula		Normal humans (male)	11	30 y/o	[116]
7.	Topical applications	20 *μ*L of 0.1% (about 0.67 mg/kg body weight) apigenin	Ethanol	Chrysanthemum		Hairless mice (female)	-	6–8 weeks old	[117]
8.	Topical formulation	60 μl of 2% hesperidin	Ethanol	Orange (Peel)		Hairless mice	-	6–8 weeks old	[118]
						Fed mouse diet (female)			
9.	Essential oil	3% w/w essential oil of *R. alba*	N/A	Rose		Wistar rats (Male)	-	11 weeks old	[119]
						Normal humans (women)	14	21.0 ± 0.1 y/o	
10.	Lotion	0.1%, 0.05% and 0.01% (v/v of 1% Eucalyptus extract)	Ethanol:water	Eucalyptus		Normal humans (Female & male)	18	33 y/o	[120]
			Methanol:water						
11.	Essence & serum	N/A	N/A	Prinsepia utilis and purslane		Acne vulgaris patients	83	Not indicate	[121]
12.	Powder (orally administered)	N/A	N/A	Radix rehmanniae, radix scrophulariae, radix ophiopogonis, poria, rhizoma dioscoreae, fructus corni, rhizoma alismatis, radix paeoniae alba, cortex moutan		Dermatitis patients	100	Not indicate	[122]
13.	Topical formulation	6.0% w/w of Camellia sinensis glycolic leaf extract	N/A	Camellia sinensis (leaf)		Normal humans (Female)	24	25–40 y/o	[123]
14.	Topical formulation	0.1% apigenin	N/A	Chrysanthemum		Hairless mice (Female)	-	6–8 weeks old	[124]
15.	Cream	Different concentrations (0–15.00 % w/w)	water/propylene glycol	Seaweed thalli		Normal humans (Female)	10	27 y/o	[125]
16.	Cream	3% of *M. oleifera* leaf extract	N/A	Moringa (leaves)		Normal humans (male)	11	20-35 y/o	[126]
17.	Cream	N/A	Organic solvent	Terminalia arjuna		Postmenopausal patients (Female)	60	50–70 y/o	[127]
18.	Cream	Different concentrations (4–5 % w/w)	N/A	Pomegranate seed oil, grape seed oil, sesame oil, flower honey		Normal humans (Female)	12	25-65 y/o	[128]
19.	Cream	0.5% *C. indicum* extract	Methanol	Chrysanthemum indicum (flowers)		Normal humans (Female)	30	41–50 y/o	[129]
20.	Powder (orally administered)	N/A	Ethanol:water	Panax ginseng Meyer		Normal humans (Female)	98	40-60 y/o	[130]
21.	Cream	N/A	N/A	Panax ginseng and Crataegus pinnatifida		Normal humans (Female)	21	30-65 y/o	[131]
22.	Topical formulation	N/A	Ethanol	Panax ginseng Meyer		Hairless mice	32	6 weeks old	[132]

## 6 Conclusion

This paper reviewed several non-invasive techniques to examine the skin physiological conditions, epidermal lipid profiles, and evaluation of herbal-based product efficiency. The advantages and limitations of each of the methods are briefly discussed. Although the marketplace is flooded with a diverse array of commercial skin analysis tools, it is important to use the most appropriate techniques to measure skin properties depending on the skin issues. The development of advanced non-invasive diagnostic techniques allows tissue imaging *in vivo* and contributes to a more accurate diagnosis of skin diseases.

In recent years, there has been an increasing interest on non-invasive techniques in clinical and investigational dermatology. The classical methods have substantially improved, leading to the development of novel tools and provide a growing number of biophysical methods to assess skin properties. The availability of non-invasive techniques in dermatology shows substantial differences concerning their limitations and opportunities, potential clinical applicability and practicability. Future research should aim to improve the technical limitations and investigate the impact of combining two or more techniques in order to enhance the diagnostic impact.

## Declarations

### Author contribution statement

All authors listed have significantly contributed to the development and the writing of this article.

### Funding statement

This work was supported by the HICOE research grant (R.J130000.7846.4J266), Ministry of Higher Learning Institution of Malaysia.

### Competing interest statement

The authors declare no conflict of interest.

### Additional information

No additional information is available for this paper.

## References

[1] Rogiers V., Balls M., Basketter D., Berardesca E., Edwards C., Elsner P. (1999). The potential use of non-invasive methods in the safety assessment of cosmetic products. Alternative Lab Anim..

[2] Di Nardo A.D., Wertz P., Giannetti A., Seidenari S. (1998). Ceramide and cholesterol composition of the skin of patients with atopic dermatitis. Acta Derm. Venereol..

[3] Feingold K.R. (2009). The outer frontier: the importance of lipid metabolism in the skin. J. Lipid Res..

[4] Reilly R.M., Ferdinando D., Johnston C. (1997). The epidermal nerve fibre network: characterization of nerve fibres in human skin by confocal microscopy and assessment of racial variations. Br. J. Dermatol..

[5] Gonzalez S., Gilaberte-Calzada Y. (2008). In vivo reflectance-mode confocal microscopy in clinical dermatology and cosmetology. Int. J. Cosmet. Sci..

[6] Rajadhyaksha M., Gonzalez S., Zavislan J., Andersn R.R., Webb R.H. (1995). In vivo confocal scanning laser microscopy of human skin: melanin provides strong contrast. J. Invest. Dermatol..

[7] Segre J.A. (2006). Epidermal barrier formation and recovery in skin disorders. J. Clin. Invest..

[8] Kapoor V. (2005). Herbal cosmetics for skin and hair care. Indian J. Nat. Prod. Resour..

[9] Bateman J., Chapman R., Simpson D. (1998). Possible toxicity of herbal remedies. Scot. Med. J..

[10] Elvin-Lewis M. (2001). Should we be concerned about herbal remedies. J. Ethnopharmacol..

[11] Murphy J.M. (1999). Preoperative considerations with herbal medicines. AORN.

[12] Hoareau L., DaSilva E.J. (1999). Medicinal plants: a re-emerging health aid. Electron. J. Biotechnol..

[13] Kanba S., Yamada K., Mizushima H., Asai M. (1998). Use of herbal medicine for treating psychiatric disorders in Japan. Psychiatr. Clin. Neurosci..

[14] Vogel H.G. (1991). Similarities between various systems of traditional medicine. Considerations for the future of ethnopharmacology. J. Ethnopharmacol..

[15] Wong A.H., Smith M., Boon H.S. (1998). Herbal remedies in psychiatric practice. Arch. Gen. Psychiatr..

[16] Boundless “Structure of the skin: epidermis.” boundless anatomy and physiology boundless. https://www.boundless.com/physiology/textbooks/boundless-anatomy-and-physiology-textbook/integumentary-system-5/the%20skin-64/structure-of-the-skin-epidermis-394-7794.

[17] Marks J., Miller G. (2006). Lookingbill and Marks' Principles of Dermatology.

[18] Proksch E., Brandner J.M., Jensen J.M. (2008). The skin: an indispensable barrier. Exp. Dermatol..

[19] Madison K.C. (2003). Barrier function of the skin: “la raison d’être” of the epidermis. J. Invest. Dermatol..

[20] Grice E.A., Kong H.H., Conlan S., Deming C.B., Davis J., Young A.C. (2009). Topographical and temporal diversity of the human skin microbiome. Science.

[21] Fluhr J.W., Feingold K.R., Elias P.M. (2006). Transepidermal water loss reflects permeability barrier status: validation in human and rodent in vivo and ex vivo models. Exp. Dermatol..

[22] Fluhr J.W., Kao J., Jain M., Ahn S.K., Feingold K.R., Elias P.M. (2001). Generation of free fatty acids from phospholipids regulates stratum corneum acidification and integrity. J. Invest. Dermatol..

[23] Atrux-Tallau N., Huynh N.T., Gardette L. (2008). Effects of physical and chemical treatments upon biophysical properties and micro-relief of human skin. Arch. Dermatol. Res..

[24] Endo K., Suzuki N., Yoshida O. (2007). The barrier component and the driving force component of transepidermal water loss and their application to skin irritant tests. Skin Res. Technol..

[25] Chamlin S.L., Kao J., Frieden I.J. (2002). Ceramide-dominant barrier repair lipids alleviate childhood atopic dermatitis: changes in barrier function provide a sensitive indicator of disease activity. J. Am. Acad. Dermatol..

[26] Rim J.H., Jo S.J., Park J.Y. (2005). Electrical measurement of moisturizing effect on skin hydration and barrier function in psoriasis patients. Clin. Exp. Dermatol..

[27] Angelova-Fischer I., Bauer A., Hipler U.C., Petrov I., Kazandjieva J., Bruckner T., Diepgen T., Tsankov N., Williams M., Fischer T.W., Elsner P., Fluhr J.W. (2005). The objective severity assessment of atopic dermatitis (OSAAD) score: validity, reliability and sensitivity in adult patients with atopic dermatitis. Br. J. Dermatol..

[28] Fluhr J.W., Darlenski R., Angelova-Fischer I. (2008). Skin irritation and sensitization: mechanisms and new approaches for risk assessment. Skin Pharmacol. Physiol..

[29] Rogiers V. (2001). EEMCO guidance for the assessment of transepidermal water loss in cosmetic sciences. Skin Pharmacol. Appl. Skin Physiol..

[30] (2017). Clinical Testing of Cosmetic and Skin Care Products Methods and Instrumentations.

[31] (2003). Information and Operating Instruction for Multi Skin Centre®.

[32] Pinnagoda J., Tupker R.A., Agner T. (1990). Guidelines for transepidermal water loss (TEWL) measurement. A report from the standardization group of the European society of contact dermatitis. Contact Dermatitis.

[33] Barel A., Clarys P., Serup J., Jemec G., Grove G. (2006). Measurement of Epidermal Capacitance.

[34] (2017). Courage Khazaka.

[35] Muehlenbein Michael PM. (2010). Human Evolutionary Biology.

[36] (2017). Courage Khazaka.

[37] Sue E.H., Kathryn L.M. (2016). Understanding Pathophysiology: Biology, Human Biology, Cram101 Textbook Reviews.

[38] Ryu H.S., Joo Y.H., Kim S.O., Park K.C., Youn S.W. (2008). Influence of age and regional differences on skin elasticity as measured by the Cutometer®. Skin Res. Technol..

[39] (2017). Courage Khazaka.

[40] Lodish H., Berk A., Zipursky S.L. (2000). Collagen: the Fibrous Proteins of the Matrix, Molecular Cell Biology.

[41] Bleve M., Priscilla C., Franca P., Paola P. (2012). Ultrasound and 3D skin imaging: methods to evaluate efficacy of striae distensae treatment. Dermatol. Res. Prat..

[42] O’goshi K. (2006).

[43] (2017). Courage Khazaka.

[44] Munke S., Aßmus U., Banowski B., Blaak J., Brock M., Erasmy J., Wood C. (2013). The impact of cleansing products on the skin surface pH. Int. Fed. Soc. Cos. Chem..

[45] Parra J.L., Paye M. (2003). EEMCO guidance for the in vivo assessment of skin surface pH. Skin Pharmacol. Appl. Skin Physiol..

[46] (2017). Courage Khazaka.

[47] Kondo H., Ichikawa Y., Imokawa G. (1998). Percutaneous sensitization with allergens through barrier-disrupted skin elicits a Th2-dominant cytokine response. Eur. J. Immunol..

[48] Bommannan D., Potts R.O., Guy R.H. (1990). Examination of stratum corneum barrier function in vivo by infrared spectroscopy. J. Invest. Dermatol..

[49] Guz N.V., Gaikwad R.M., Dokukin M.E., Sokolov I. (2009). A novel in vitro stripping method to study geometry of corneocytes with fluorescent microscopy: example of aging skin. Skin Res. Technol..

[50] Schwarb F.P., Gabard B., Rufli T., Surber C. (1999). Percutaneous absorption of salicylic acid in man after topical administration of three different formulations. Dermatology.

[51] Cambon M., Issachar N., Castelli D., Robert C. (2001). An in vivo method to assess the photostability of UV filters in a sunscreen. J. Cosmet. Sci..

[52] Surber C., Wilhelm K.P., Bermann D., Maibach H.I. (1993). In vivo skin penetration of acitretin in volunteers using three sampling techniques. Pharm. Res. (N. Y.).

[53] Wagner H., Kostka K.H., Lehr C.M., Schaefer U.F. (2001). Interrelation of permeation and penetration parameters obtained from in vitro experiments with human skin and skin equivalents. J Control Release.

[54] Benech-Kieffer F., Wegrich P., Schwarzenbach R., Kleca G., Weber T., Leclaire J., Schaefer H. (2000). Percutaneous absorption of sunscreens in vitro: interspecies comparison, skin models and reproducibility aspects. Skin Pharmacol. Appl. Skin Physiol..

[55] Potard G., Laugel C., Schaefer H., Marty J.P. (2000). The stripping technique: in vitro absorption and penetration of five UV filters on excised fresh human skin. Skin Pharmacol. Appl. Skin Physiol..

[56] Ya-Xian Z., Suetake T., Tagami H. (1999). Number of cell layers of the stratum corneum in normal skin – relationship to the anatomical location on the body, age, sex and physical parameters. Arch. Dermatol. Res..

[57] Black D., Del Pozo A., Lagarde J.M., Gall Y. (2000). Seasonal variability in the biophysical properties of stratum corneum from different anatomical sites. Skin Res. Technol..

[58] Schwindt D.A., Wilhelm K.P., Maibach H.I. (1998). Water diffusion characteristics of human stratum corneum at different anatomical sites in vivo. J. Invest. Dermatol..

[59] Greene R.S., Downing D.T., Pochi P.E., Strauss J.S. (1970). Anatomical variation in the amount and composition of human skin surface lipid. J. Invest. Dermatol..

[60] De Jongh C.M., Verberk M.M., Spiekstra S.W., Gibbs S., Kezic S. (2007). Cytokines at different stratum corneum levels in normal and sodium lauryl sulphate-irritated skin. Skin Res. Technol..

[61] Jungersted J.M., Hellgren L.I., Jemec G.B.E., Agner T. (2008). Lipids and skin barrier function - a clinical perspective. Contact Dermatitis.

[62] Weerheim A., Ponec M. (2001). Determination of stratum corneum lipid profile by tape stripping in combination with high performance thin-layer chromatography. Arch. Dermatol. Res..

[63] Norle´n L., Nicander I., Rozell B.L., Ollmar S., Foslind B. (1999). Inter- and intra-individual differences in human stratum corneum lipid content related to physical parameters of skin barrier function in vivo. J. Invest. Dermatol..

[64] Denda M., Hori J., Koyama J., Yoshida S., Nanba R., Takahashi M., Horii I., Yamamoto A. (1992). Stratum corneum sphingolipids and free amino acids in experimentally-induced scaly skin. Arch. Dermatol. Res..

[65] Dreher F., Arens A., Hostynek J.J. (1998). Colorimetric method for quantifying human stratum corneum removed by adhesive-tape stripping. Acta Derm. Venereol..

[66] Dreher F., Modjtahedi B.S., Modjtahedi S.P. (2005). Quantification of stratum corneum removal by adhesive tape stripping by total protein assay in 96-well microplates. Skin Res. Technol..

[67] Jacobi U., Kaiser M., Koscielny J., Schutz R., Meinke M., Sterry W., Lademann J. (2006). Comparison of blood flow to the cutaneous temperature and redness after topical application of benzyl nicotinate. J. Biomed. Optic..

[68] Jacobi U., Toll R., Audring H., Sterry W., Lademann J. (2005). The porcine snout – an in vitromodel for human lips. Exp. Dermatol..

[69] Bashir S.J., Chew A.L., Anigbogu A. (2001). Physical and physiological effects of stratum corneum tape stripping. Skin Res. Technol..

[70] Lademann J., Jacobi U., Surber C., Weigmann H.J., Fluhr J.W. (2009). The tape stripping procedure – evaluation of some critical parameters. Eur. J. Pharm. Biopharm..

[71] Burgdorf W. (2005). Ultrasound scanning in dermatology. Arch. Dermatol..

[72] Scrupt J., Keiding J., Fullerton A., Gniadecka M., Gniadecki R. (1995). High Frequency Ultrasound Examination of Skin; Introduction and Guide, Handbook of Non-invasive Methods and the Skin.

[73] Thiboutot D.M. (1999). Dermatological applications of high-frequency ultrasound. Medical Imaging—Ultrasonic Transducer Engineering, vol. 3664 of Proceedings of SPIE.

[74] Schmid-Wendtner M.H., Burgdorf W. (2005). Ultrasound scanning in dermatology. Arch. Dermatol..

[75] Bielfeldt S., Buttgereit P., Brandt M., Springmann G., Wilhelm K.P. (2008). Non-invasive evaluation techniques to quantify the efficacy of cosmetic anti-cellulite products. Skin Res. Technol..

[76] El-Gammal S., Hoffman K., Auer T., Korten M., Altmeyer P., Hoss A., Erment H. (1992). A 50-MHz High Resolution Ultrasound Imaging System from Dermatology; Ultrasound in Dermatology.

[77] Elsner P. (1998).

[78] Wortsman X., Wortsman J. (2010). Clinical usefulness of variable-frequency ultrasound in localized lesions of the skin. J. Am. Acad. Dermatol..

[79] Fornage B.D., McGavran M.H., Duvic M. (1993). Imaging of the skinwith 20- MHz US. Radiology.

[80] Desai T.D., Desai A.D., Horowitz D.C. (2007). The use of high-frequency ultrasound in the evaluation of superficial and nodular basal cell carcinomas. Dermatol. Surg..

[81] Kleinerman R., Whang T.B., Bard R.L., Marmur E.S. (2012). Ultrasound in dermatology: principles and applications. J. Am. Acad. Dermatol..

[82] New K.C., Petroll W.M., Boyde A., Martin L., Corcuff P., Leveque J.L., Lemp M.A., Cavanagh H.D., Jester J.V. (1991). In vivo imaging of human teeth and skin using real time confocal microscopy. Scanning.

[83] Paul D.W., Ghassemi P., Ramella-Roman J.C., Prindeze N.J., Moffatt L.T., Alkhalil A. (2015). Noninvasive imaging technologies for cutaneous wound assessment: a review. Wound Repair Regen..

[84] Corcuff P., Bertrand C., Leveque J.L. (1993). Morphometry of human epidermis in vivo by real time confocal microscopy. Arch. Dermatol. Res..

[85] Anderson R.R., Parrish J.A. (1983). Selective photothermolysis: precise microsurgery by selective absorption of pulsed radiation. Science.

[86] Haina D., Landthaler M., Braun-Falco O., Waidelich W. (1987). Comparison of the maximum coagulation depth in skin for different types of medical lasers. Laser Surg. Med..

[87] Goldman L., Younger B., Peny E. (1982). Surgery with the argon laser: recent advances in instrumentation and techniques. Dermatol. Surg..

[88] Spicer S., Goldberg D.J. (1996). Lasers in dermatology. J. Am. Acad. Dermatol..

[89] Shokrollahi K., Raymond E., Murison M.S.C. (2004). Lasers : principles and surgical applications. J. Surg..

[90] Rajadhyaksha M., Gonzalez S., Zavislan J.M., Anderson R.R., Webb R.H. (1999). In vivo confocal scanning laser microscopy of human skin II: advances in instrumentation and comparison with histology. J. Invest. Dermatol..

[91] Terhorst D., Maltusch A., Stockfleth E., Lange-Asschenfeldt S., Sterry W., Ulrich M. (2011). Reflectance confocal microscopy for the evaluation of acute epidermal wound healing. Wound Repair Regen..

[92] Pierard G.E., Arrese J.E., Pierre S., Bertrand C., Corcuff P., Leveque J.L., Pierard C. (1994). Diagnosis microscopic of onychomychoses. Ann. Dermatol. Venererol..

[93] Corcuff P., Gonnord G., Pierard G.E., Leveque J.L. (1996). In vivo confocal microscopy of human skin; a new design for cosmetology and dermatology. Scanning.

[111] Kapoor S., Saraf S. (2010). Assessment of viscoelasticity and hydration effect of herbal moisturizers using bioengineering techniques. Phcog. Mag..

[112] Kapoor S., Saraf S. (2010). Formulation and evaluation of moisturizer containing herbal extracts for the management of dry skin. Phcog. J..

[113] Belo S.E.D., Gaspar L.R., Campos P.M. (2011). Photoprotective effects of topical formulations containing a combination of Ginkgo biloba and green tea extracts. Phytother Res..

[114] Rasul A., Akhtar N. (2011). Formulation and in vivo evaluation for anti-aging effects of an emulsion containing basil extract using non- invasive biophysical techniques. Daru: J. Facul. Pharm..

[115] Funakushi N., Yamaguchi T., Jiang J., Imamura S., Kuhara T., Suto H., Ikeda S. (2011). Ameliorating effect of Yokukansan on the development of atopic dermatitis-like lesions and scratching behavior in socially isolated NC/Nga mice. Arch. Dermatol. Res..

[116] Akhtar N., Khan A.B., Muhammad S. (2012). Formulation and characterization of a cream containing Terminalia chebula extract. Forsch Komplementmed.

[117] Man M.Q., Hupe M., Sun R., Man G., Mauro T.M., Elias P.M. (2012). Topical apigenin alleviates cutaneous inflammation in murine models. Evid. base Compl. Alternative Med.: eCAM.

[118] Hou M., Man M., Man W., Zhu W., Hupe M., Park K., Man M.Q. (2012). Topical hesperidin improves epidermal permeability barrier function and epidermal differentiation in normal murine skin. Exp. Dermatol..

[119] Fukada M., Kano E., Miyoshi M., Komaki R., Watanabe T. (2012). Effect of “rose essential oil” inhalation on stress-induced skin-barrier disruption in rats and humans. Chem. Senses.

[120] Ishikawa J., Shimotoyodome Y., Chen S., Ohkubo K., Takagi Y., Fujimura T., Takema Y. (2012). Eucalyptus increases ceramide levels in keratinocytes and improves stratum corneum function. Int. J. Cosmet. Sci..

[121] Pang Q., Tu Y., He L. (2012). Clinical study on effects of skin care products containing extract of prinsepia utilis and purslane in the treatment of acne vulgaris. J. Clin. Dermatol..

[122] Peng H.H. (2013). Clinical research on mechanism of “Jia-Jian Xiao-Yao Powder” improving facial skin barrier function of hormone-dependent dermatitis patients. World Sci. Technol.-Modern Tradit. Chin. Med..

[123] Gianeti M.D., Mercurio D.G., Maia Campos P.M.B.G. (2013). The use of green tea extract in cosmetic formulations: not only an antioxidant active ingredient. Dermatol. Ther..

[124] Hou M., Sun R., Hupe M., Kim P.L., Park K., Crumrine D., Man M.Q. (2013). Topical apigenin improves epidermal permeability barrier homeostasis in normal murine skin by divergent mechanisms. Exp. Dermatol..

[125] Choi J.S., Moon W.S., Choi J.N. (2013). Effects of seaweed Laminaria japonica extracts on skin moisturizing activity in vivo. J. Cosmet. Sci..

[126] Ali A., Akhtar N., Chowdhary F. (2014). Enhancement of human skin facial revitalization by moringa leaf extract cream. Adv Dermatol Allergol/Postḝpy Dermatologii I Alergologii.

[127] Farwick M., Köhler T., Schild J. (2014). Pentacyclic triterpenes from Terminalia arjuna show multiple benefits on aged and dry skin Skin. Pharmacol. Phys..

[128] Altuntaş E., Yener G. (2015). Anti-aging potential of a cream containing herbal oils and honey: formulation and in vivo evaluation of effectiveness using non- invasive biophysical techniques. IOSR J. Pharm. Biol. Sci. Ver. I.

[129] Choi K.T., Kim J.H., Cho H.T., Lim S.S., Kwak S.S., Kim Y.J. (2016). Dermatologic evaluation of cosmetic formulations containing Chrysanthemum indicum extract. J. Cosmet. Dermatol..

[130] Park S.Y., Shin Y.K., Kim H.T., Kim Y.M., Lee D.G., Hwang E., Yi T.H. (2016). A single-center, randomized, double-blind, placebo-controlled study on the efficacy and safety of “enzyme-treated red ginseng powder complex (BG11001)” for antiwrinkle and proelasticity in individuals with healthy skin. J. Ginseng Res..

[131] Hwang E., Park S.Y., Yin C.S., Kim H.T., Kim Y.M., Yi T.H. (2017). Antiaging effects of the mixture of Panax ginseng and Crataegus pinnatifida in human dermal fibroblasts and healthy human skin. J. Ginseng Res..

[132] Hong Y.H., Lee H.S., Jung E.Y., Han S.H., Park Y., Suh H.J. (2017). Photoprotective effects of topical ginseng leaf extract using Ultraflo L against UVB-induced skin damage in hairless mice. J. Ginseng Res..

